# Multi-Omics Integration Reveals Electroacupuncture Ameliorates Cognitive Impairment in Alzheimer’s Disease via Gut–Brain Axis

**DOI:** 10.3390/biom15111486

**Published:** 2025-10-22

**Authors:** Shuai Zhang, Xinyuan Liu, Shuyu Xu, Weixian Li, Jie Song, Qing Tian, Yanjun Du

**Affiliations:** 1 College of Acupuncture-Moxibustion and Orthopaedics, Hubei University of Chinese Medicine, Wuhan 430061, China; 2 Department of Pathology and Pathophysiology, School of Basic Medicine, Tongji Medical College, Huazhong University of Science and Technology, Wuhan 430074, China; 3 Hubei International Science and Technology Cooperation Base of Preventive Treatment by Acupuncture and Moxibustion, Wuhan 430061, China; 4 Hubei Shizhen Laboratory, Wuhan 430061, China

**Keywords:** Alzheimer’s disease, 16S rRNA sequencing, multi-omics integration, electroacupuncture, gut–brain axis

## Abstract

Background: Alzheimer’s disease (AD) lacks effective therapeutic strategies. Electroacupuncture (EA) offers promising neuroprotective effects, but its underlying mechanisms remain unclear. Objective: To explore the mechanisms of EA’s neuroprotective effects on AD via microbiome and metabolome integration. Methods: Utilizing a well-established model of AD, Senescence-Accelerated Mouse Prone 8 (*SAMP8*), EA intervention was performed. 16S ribosomal RNA (rRNA) sequencing and serum metabolomics were conducted on *SAMP8* mice, *SAMP8* mice after EA intervention, and their normal control group Senescence-Accelerated Mouse Resistant 1 (*SAMR1*) mice. Results: *SAMP8* mice were subjected to electroacupuncture (EA) treatment at the Baihui (GV20) and Shenshu (BL23) acupoints for 15 min daily over a period of four weeks. EA enhanced cognitive function and reduced neuronal damage in AD models. The treatment lowered pro-inflammatory cytokines (TNF-α, IL-1β) and AD-related pathologies (tau, Aβ_1-42_). EA also rebalanced gut microbiota by increasing beneficial *Gastranaerophilales* while decreasing harmful *Proteobacteria*. Additionally, it restored purine and phenylpropanoid metabolism by regulating key metabolites. Importantly, EA reduced levels of specific metabolites linked to pro-inflammatory bacteria (*Sphingomonas, Massilia, Escherichia-Shigella*), simultaneously decreasing their abundance. These findings highlight EA’s multi-target effects on neuroinflammation, gut microbiota, and metabolic pathways in AD. Notably, the interactions between EA-regulated key metabolites and AD-related targets, predicted via PubChem and ChEMBL databases, remain computational and have not been validated by experimental studies. Conclusions: EA exerts neuroprotective effects in AD via modulation of gut microbiota and metabolic pathways, representing a novel non-pharmacological therapeutic strategy.

## 1. Introduction

Alzheimer’s disease (AD), the predominant form of dementia, characterized by progressive cognitive decline, has emerged as a critical global public health challenge [1]. The etiology of AD is intricate and diverse, with its precise mechanisms underlying its onset remaining incompletely understood. In addition to the core pathologies of amyloid-β (Aβ) and tau, multiple factors may also be involved in the pathological process of AD, including acetylcholine deficiency, neuroinflammation, oxidative stress, glutamate imbalance, mitochondrial dysfunction, and autophagic abnormalities, gut microbiota abnormalities [2].

Microbiota play a pivotal role in regulating physiological functions in both health and disease contexts. Previous studies have demonstrated the communication between gut microbiota and the brain, named as the gut–brain axis [3]. Notably, recent studies have shown that the composition of the gut microbiota is altered by increased gut barrier permeability, which in turn impairs blood–brain barrier function. This cascade mediated by the gut–brain axis promotes neuroinflammation, neuronal loss, neural injury, and ultimately contributes to the development of AD [4]. Therefore, dysbiosis of the gut microbiota is recognized as a critical factor driving neuroinflammation through the gut–brain axis, ultimately leading to neuronal damage and cognitive decline [5]. For instance, an animal study has demonstrated that fecal microbiota transplantation (FMT) from AD mice into wild-type mice can induce memory impairment and neuroinflammation [6]. Furthermore, the composition and relative abundance of gut bacteria from phylum to genus are different in AD patients compared with healthy people [7].

Cognitive decline has been associated with a decrease in gut microbiota diversity, which is characterized by a reduction in the diversity of beneficial bacteria, such as Lactobacillus and Bifidobacteria, and an increase in the number of pro-inflammatory bacteria, including Propionibacteria, Fusobacteria, Shigella, and Clostridia [8]. Among these, short-chain fatty acids (SCFAs) derived from gut microbiota metabolism play a pivotal role in the initiation and progression of AD. For example, acetate, an SCFA produced by gut bacteria, promotes microglial maturation, regulates homeostatic metabolic states, and modulates AD pathology by influencing microglial innate immune mechanisms [9]. In summary, the gut microbiota is involved in multiple potential mechanisms underlying the pathological development of AD. However, the strains that play a dominant role in neurological diseases still need to be explored. Therefore, further exploration of the complex gut–brain axis network is essential to develop interventions and treatments for AD targeting the gut microbiota.

Acupuncture therapy, a traditional Chinese medicine (TCM) practice first documented in the Huangdi Neijing (Inner Canon of the Yellow Emperor), involves inserting needles into specific acupoints to treat various diseases. With a history spanning thousands of years, acupuncture is celebrated for its beneficial therapeutic effects and minimal side effects. Electroacupuncture (EA) is widely employed in clinical practice. Preclinical studies have shown that EA exerts multiple effects, including analgesia, muscle relaxation, anti-inflammatory, mild anxiolytic, and antidepressant actions [10,11]. Its biological mechanisms may involve central sensitization modulation, neurotransmitter regulation, gut microbiota modulation, immune system adjustment, oxidative stress reduction, and neuroinflammatory suppression [12]. For AD, clinical study has demonstrated that EA improves cognitive function and other clinical outcomes in AD patients without significant adverse effects [13]. Additionally, EA can alleviate cognitive impairment in AD by mitigating neuroinflammation, enhancing synaptic plasticity, inhibiting nerve cell apoptosis, and reducing the production and aggregation of Aβ in the brain [14,15].

In recent years, EA, a modernized form of traditional acupuncture, has demonstrated significant potential in ameliorating neurodegenerative diseases due to its systemic regulatory effects and favorable safety profile [16]. Studies have shown that EA can restore gut microbiota balance, rectify blood–brain barrier dysfunction, and mitigate neuroinflammation [17,18,19]. For instance, in AD models, EA significantly increased the abundance of Bifidobacterium and Lactobacillus while suppressing the overgrowth of Proteobacteria and reducing pro-inflammatory cytokine levels [20]. Furthermore, EA modulates neuroinflammatory signaling pathways such as TLR4/NF-κB and NLRP3/caspase-1 and enhances synaptic plasticity and antioxidant capacity by activating genes like brain-derived neurotrophic factor (BDNF) [,^21^]. Concurrently, metabolomic studies reveal that EA improves metabolic disturbances by regulating key pathways, including glycolysis, purine metabolism, and the tricarboxylic acid cycle-thereby exerting neuroprotective effects [22]. Nevertheless, the precise mechanisms by which EA influences specific central nervous system targets through gut microbiota and metabolites remain incompletely understood.

However, comprehensive studies integrating multi-omics approaches to systematically decipher the molecular mechanisms underlying EA’s therapeutic efficacy in AD remain limited. In this work, we utilized integrative microbiomics and metabolomics to explore the complex interplay between gut microbiota and host metabolites following EA treatment in AD mouse model. Furthermore, we depleted the gut microbiota of AD mice model using antibiotic pretreatment before EA intervention. We utilized classic behavioral assays, including the Morris water maze and novel object recognition tests, as well as ELISA analyses, to validate the mediating role of the gut microbiota in EA-induced neuroprotection. Collectively, our study aims to provide a foundation for developing EA as a precision therapeutic strategy in AD management from a multi-omics perspective.

## 2. Material and Methods

### 2.1. Animals and Experimental Groups

Senescence-Accelerated Mouse Prone 8 (*SAMP8*) and Senescence-Accelerated Mouse Resistant (*SAMR1*) aged 24 weeks were purchased from Peking University Health Science Center (Beijing, China). All the animals were housed at a temperature of 22 ± 1 °C, relative humidity of 50 ± 1%, and a light/dark cycle of 12/12 h; they were fed with sterile food and water ad libitum. Prior to formal experiments, mice with cognitive impairment were excluded through screening via the novel object recognition test.

The animals were randomly divided into four groups, and each group contained 12 male mice: (1) Control group: Consisted of *SAMR1* that received simultaneous restraint with the EA group, without any additional intervention (n = 12). (2) Model group: Consisted of *SAMP8* that received simultaneous restraint with the EA group, without any additional intervention (n = 12). (3) EA group: Consisted of *SAMP8* underwent electroacupuncture treatment for 4 weeks (n = 12). Specific protocols were as follows: underwent electroacupuncture at bilateral GV20 and BL23 according to a widely recognized mouse acupoint atlas [1]. The location of the three acupoints was also clearly described in a systematic review of animal-based studies on the effects of acupuncture on AD [2]. For the GV20 acupoint (Baihui), acupuncture needles (specification: 0.16 mm × 7 mm) manufactured by Universal Acupuncture Medical Equipment Co., Ltd. (Suzhou, China) were inserted horizontally at a 15-degree angle to a depth of 1–2 mm beneath the skin/acupoint. For the BL23 acupoint (Shenshu), needles were inserted perpendicularly (90 degrees) to a depth of 2–3 mm. Subsequently, the needles were connected to the Han’s electroacupuncture apparatus (HANS-100A, Nanjing Jisheng Medical Technology Co., Ltd., Nanjing, China). Electroacupuncture parameters were set to continuous wave mode with a current intensity of 1 mA and a frequency of 2 Hz. The stimulation intensity was adjusted to induce a slight tremor of the needle handle while ensuring the mouse exhibited no struggling or vocalization. Daily electroacupuncture intervention was administered once per day by operators who had received standardized training prior to the study. Each treatment course lasted 6 consecutive days. Mice in the treatment group received a total of four treatment courses, with one-day intervals between courses. (4) EA+Antibiotic group: Antibiotics were administered to *SAMP8* via drinking water (ampicillin 1 mg/mL, metronidazole 1 mg/mL, neomycin 1 mg/mL, vancomycin 0.5 mg/mL. All the above antibiotics were purchased from Sigma-Aldrich Co., Ltd., St. Louis, MO, USA) for 2 weeks before EA intervention in the EA+Antibiotic group, and discontinued during EA treatment (n = 12). The control, Model, and EA groups received normal sterile water without antibiotics.

Mice were housed in individual ventilated cages with 3 mice per cage, and bedding was changed every 3 days. Restraint was applied for 15 min daily (consistent with EA treatment duration) in the Control and Model groups to match the stress level of the EA and EA+Antibiotic groups.

Water intake was recorded daily by measuring the volume of water consumed per cage, and body weight was measured weekly. No significant differences in water intake (mean 4–5 mL/mouse/day) or body weight (stable at 28–32 g) were observed among groups during the experiment, indicating that antibiotics did not affect water intake or general health of the mice.

### 2.2. Ethical Review

The detailed information including background, research purpose, study design, and experimental methods were submitted during application process. The experimental protocol was approved (HUCMS-00312778) by the Animal Ethics Committee of the Hubei University of Chinese Medicine, and animal experiments were performed in compliance with the guidelines of the Animal Ethics Committee of the University.

### 2.3. Behavioral Testing

All mice underwent the Morris water maze test (MWM) on the day following the completion of the aforementioned interventions to assess their spatial learning and memory abilities. All mice were required to complete both the place navigation test and the spatial probe test. Specifically, formal place navigation training was conducted once daily for 5 consecutive days. During each hidden platform trial, a mouse was placed into the water facing the wall of the circular black pool (diameter: 120 cm, height: 50 cm) from one of four entry points located in different quadrants. The time taken for the mouse to find and climb onto the hidden platform in the fixed quadrant within 1 min was recorded as the escape latency (in seconds), serving as the behavioral performance indicator. If a mouse failed to reach the target platform within 1 min, the escape latency was recorded as 60 s by default. Four platform trials were performed per mouse each day to obtain an average escape latency value. On the day following the completion of place navigation training, the spatial probe test was conducted. The target platform was removed, and the mouse was placed into the water from any entry point in a quadrant other than the original platform quadrant. The number of times the mouse crossed the original platform site within 1 min was recorded. Throughout the entire experiment, data recording and analysis were performed using the WMT-100 Morris video tracking system (Chengdu Taimeng Technology Co., Ltd., Chengdu, China).

After the MWM test, the novel object recognition test (NOR) was performed. The NOR test was used to evaluate short-term memory and performed as previously described. At first, animals were placed in an open arena along with two identical objects situated 15 cm from the side walls in two opposite parts of the maze. Animals were left to freely explore the arena and the object for 6 min before being placed back in their cage. After 60 min, a novel object was placed instead of one of the two identical initial objects. The animals were then given an additional 6 min to explore the new arena. For each animal, the discrimination index (the percentage of the time exploring the novel object among the total time spent exploring both objects) was determined.

### 2.4. Tissue Processing and Histopathology

At the end of the behavioral test, animals were first anesthetized with sodium pentobarbital (50 mg/kg). Six animals per group were then euthanized by cervical dislocation, followed by immediate dissection of fresh hippocampal and cortical tissues on ice. The remaining six animals per group underwent transcardial perfusion with ice-cold 0.9% sodium chloride, followed by perfusion with 4% paraformaldehyde (PFA) via left ventricular catheter until cardiac arrest occurred to complete the euthanasia procedure. The mid-sagittally cut hemispheres underwent paraffin embedding, and 5 μm thick serial sections were prepared using the HM355S rotary microtome (Thermo Fisher Scientific, Walldorf, Germany), which features a waterfall-based section transfer system and Peltier cooling elements. Initial sections from each animal series were prepared and analyzed following routine Nissl staining, while consecutive serial sections were processed for immunohistochemistry.

### 2.5. Nissl Staining

Paraffin sections were dewaxed to water. Staining the slices with toluidine blue (G1032-500 mL; Servicebio, Wuhan, China), soaking the slices in dyeing solution for 5 min, washing with water, slightly differentiating with 1% glacial acetic acid (G10000218-500 mL; Servicebio, Wuhan, China), washing with tap water to stop the reaction, controlling the degree of differentiation under microscope, and drying the slices in oven after washing with tap water. Then put the slices in clean xylene and transparent for 5 min, and seal the slices with neutral gum. Observe and take pictures under a microscope. Nissl bodies of the hippocampus and cortex were dark blue with a light blue background. The counts of Nissl bodies per area (cell counts/mm^2^) were recorded.

### 2.6. Immunohistochemistry Staining

Paraffin sections were dewaxed and rehydrated after drying at 37 °C overnight. For antigen retrieval, the hydrated sections were placed in sodium citrate buffer (P0081; Beyotime, Shanghai, China) and heated with a microwave oven (Midea, Foshan, China) until boiling for 15 min, followed by natural cooling at room temperature. The sections were then incubated with 3% hydrogen peroxide solution at 37 °C for 25 min to quench endogenous peroxidase activity, followed by washing with phosphate-buffered saline buffer solution 3 times. Afterward, the sections were incubated with primary antibodies against Tau (10274-1-AP; Proteintech, Wuhan, China) with the dilution ratio of 1:50 overnight at 4 °C, followed by incubation with the horseradish peroxidase-conjugated goat anti-rabbit IgG antibody with the dilution ratio of 1:200 for 2 h. The sections were stained with the 3,3′-diaminobenzidine substrates (P0202; Beyotime, Shanghai, China) and counterstained with hematoxylin, and dehydrated with ethanol and xylene to prepare for slide mounting. The images were viewed by a bright-field microscope (Olympus VS200, Tokyo, Japan), and the staining intensity of each image was quantified using Image J 2.0 software. The integrated optical density (IOD) and its values of the positive area (IOD/area = mean optical density (MOD)) of each image were determined and obtained as representative TAU staining density. Higher mean optical density indicates stronger positive expression.

### 2.7. ELISA

TNFα, IL1β and Aβ_1-42_ concentrations in the mouse hippocampus and cortex were quantified using a sandwich enzyme-linked immunosorbent assay (ELISA). Briefly, dissected hippocampal and cortical tissues were homogenized in ice-cold phosphate-buffered saline (PBS) containing protease inhibitors. The resulting homogenates were centrifuged (10,000× *g*, 4 °C, 15 min), and the clarified supernatants were collected. Total protein concentration in each supernatant was determined using BCA assay. Subsequently, equal amounts of total protein from each sample, alongside standards for TNFα, IL1β, and Aβ_1-42_, were loaded in duplicate into their respective mouse-specific ELISA kit wells (TNFα/IL1β/Aβ_1-42_ kits, Shanghai Enzyme-linked Biotechnology Co., Ltd., China) according to the manufacturers’ protocols. Following incubation, washing, and color development steps, absorbance was measured at the appropriate wavelength. TNFα, IL1β, and Aβ_1-42_ concentrations in the samples were interpolated from the standard curve and expressed as picograms per milligram of total protein (pg/mg protein).

### 2.8. 16S rDNA Profiling of Gut Microbiota

Genomic DNA was extracted from stool samples using the TGuide S96 Magnetic Soil/Stool DNA Kit (Tiangen Biotech, Beijing, China) following the manufacturer’s protocol. The bacterial 16S rRNA gene hypervariable V3-V4 region was amplified using the primer pair 338F (5′-CCTACGGGNGGCWGCAG-3′) and 806R (5′-GACTACHVGGGTATCTAATCC-3′). PCR products were verified on an agarose gel and purified with the Omega DNA Purification Kit (Omega Inc., Norcross, GA, USA). The purified products were sequenced on the Illumina NovaSeq 6000 platform (San Diego, CA, USA) with paired-end reads (2 × 250 bp).

### 2.9. Bioinformatics Analysis of 16S Amplicon Sequencing

Bioinformatics analysis was primarily conducted using USEARCH 8 (v8.1.1861) [23]. Briefly, primers were identified and removed using USEARCH. Sequences were filtered based on single nucleotide quality with a maximum expected error rate of 0.01. The Unoise3 algorithm was then applied to denoise the data and generate amplicon sequence variants (ASVs) [24], with ASVs containing fewer than 10 reads being discarded. Species annotation of ASVs was carried out using the Silva v123 database [25] with the SINTAX algorithm implemented by USEARCH (v8.1.1861) [26] and a confidence threshold of 60%. Alpha diversity was calculated using the R package vegan (v2.8-0) [27], while beta diversity distance matrices were computed using the R package phyloseq (v1.52.0) [28]. Principal coordinates analysis (PCoA), heatmaps, and UPGMA clustering were employed for analyzing beta diversity. Additionally, the Wilcoxon rank-sum test and Linear Discriminant Analysis Effect Size (LEfSe, v1.0.8.) [29] were utilized to detect significant taxonomic differences between groups, with a significance threshold set at *p*-value < 0.05 and FDR-adjusted *p*-value < 0.2 for feature distinction. Microbial functional prediction was performed using PICRUSt2 (v2.6.2) [30].

### 2.10. UPLC-MS/MS Metabolomics Profiling

The metabolomics analysis was conducted using a Waters ACQUITY UPLC I-Class system (Waters Corporation, Milford, MA, USA) PLUS ultra-high-performance liquid chromatography system coupled with a Waters Xevo G2-XS QTOF high-resolution mass spectrometer (Waters Co., Milford, MA, USA), equipped with a Waters ACQUITY UPLC HSS T3 column (1.8 μm, 2.1 × 100 mm). The column temperature was maintained at 40 °C with a flow rate of 0.40 mL min^−1^, and the injection volume was 2 μL. In both positive and negative ion modes, the mobile phases comprised 0.1% formic acid in water (phase A) and 0.1% formic acid in acetonitrile (phase B). The gradient program (time, %B) was: 0.0 min, 5%; 0.5 min, 5%; 5.5 min, 50%; 9.0 min, 95%; 10.5 min, 95%; 12.0 min, 5% (re-equilibration at initial conditions).

For LC-MS/MS analysis, the Xevo G2-XS QTOF was operated in MSE mode using MassLynx v4.2 for simultaneous collection of precursor and fragment ion data. Each acquisition cycle alternated low-energy (off) and elevated-energy scans (10–40 eV) at 0.2 s per spectrum over m/z 50–1200. ESI source parameters were: capillary voltage +2500 V/−2000 V (positive/negative), cone voltage 30 V, source temperature 100 °C, desolvation temperature 500 °C, desolvation gas 800 L h^−1^, and cone/backflush gas 50 L h^−1^. Samples were randomized. A pooled QC sample was injected every 10 injections, and 2-chloro-L-phenylalanine (20 mg L^−1^) served as an internal standard to monitor retention-time and response stability.

### 2.11. Metabolomics Data Processing and Annotation

Raw data generated by MassLynx v4.2 were processed in Progenesis QI (v2.2, Nonlinear Dynamics, Newcastle, UK) for peak picking, retention-time alignment, and deconvolution. Features failing standard QC criteria (e.g., poor peak shape or inconsistent QC response) were excluded prior to statistics. Metabolite identification relied on the METLIN database (https://metlin.scripps.edu/, accessed on 4 June 2025) and an in-house spectral library with theoretical fragment matching. Peak areas were normalized to the total ion current (total peak area). Principal component analysis and Spearman correlation were used to assess within-group repeatability and QC performance. Identified compounds were categorized and mapped to pathways using KEGG (www.genome.jp/kegg, accessed on 4 June 2025), HMDB (http://www.hmdb.ca/), and LIPID MAPS (www.lipidmaps.org/, accessed on 4 June 2025). Differential analysis combined fold-change calculations, two-sided *t*-tests (*p* < 0.05), and VIP > 1 from OPLS-DA modeling (R package ropls) with 200 permutation tests for model validation. KEGG pathway enrichment significance was evaluated using the hypergeometric test.

### 2.12. Evidence of Metabolite-Target Interactions

Metabolite information, including the CID (Compound ID) and SMILES notation, was retrieved using the PubChem (https://pubchem.ncbi.nlm.nih.gov/, accessed on 13 June 2025) RESTful API (Application Programming Interface) [31]. The ChEMBL ID for each metabolite was obtained via the UniChem  (https://www.ebi.ac.uk/unichem/, accessed on 13 June 2025) API [32] using the corresponding CID. To gather evidence of chemical–target interactions, the PubChem API was employed to obtain interaction evidence based on the metabolite’s CID. Subsequently, the ChEMBL [33] database was utilized to retrieve the ChEMBL ID for the metabolite’s targets. The target information was further obtained from ChEMBL, filtering for targets derived from Homo sapiens (Taxonomy ID: 9606). Additionally, target predictions were made using the ChEMBL SEA (https://sea.bkslab.org/, accessed on 13 June 2025) [34] (Similarity Ensemble Approach) API based on the SMILES notation of the metabolite. Finally, the targets from these three sources were combined to form a comprehensive list of all potential targets for the metabolites under study.

### 2.13. Functional Enrichment Analysis

To identify functional categories of genes, we employed the clusterProfiler package (v4.6.2) [35], which enabled us to determine Gene Ontology (GO) terms and KEGG pathways.

### 2.14. Other Statistical Analysis

Principal Component Analysis (PCA) was performed using the R package factoextra (https://cloud.r-project.org/package=factoextra, accessed on 8 June 2025) to visualize the clustering of samples based on metabolomic and microbiomic data, with the first two principal components (PC1 and PC2) explaining the main variation in the dataset. The pheatmap package (https://cran.r-project.org/web/packages/pheatmap/index.html, accessed on 8 June 2025) in R was used to perform the clustering based on Euclidean distance. The Wilcoxon rank-sum test was used for pairwise comparisons of non-normally distributed continuous variables between groups, with a significance threshold of *p* < 0.05.

## 3. Results

### 3.1. EA Alleviated Neuroinflammation and Restored Impaired Cognitive Functions of AD Model

**Figure 1:**
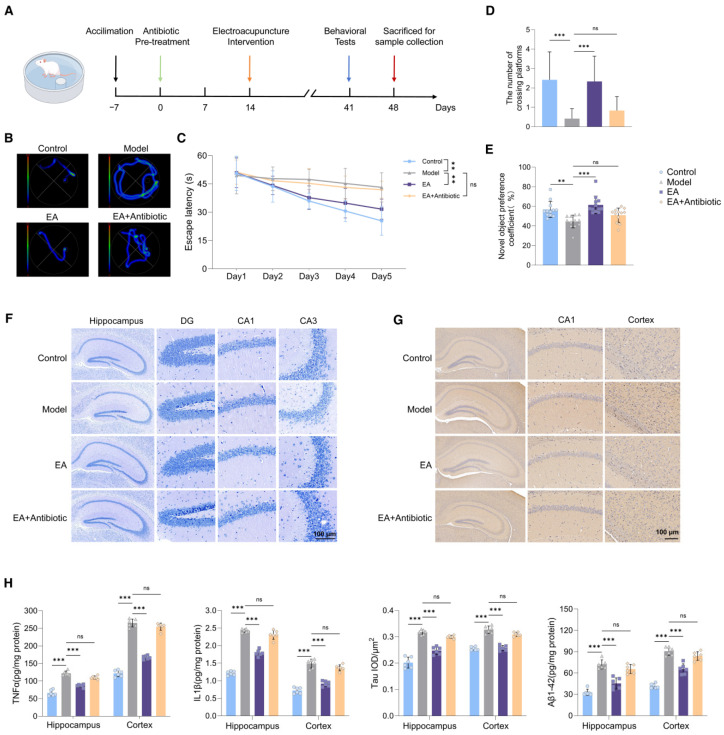
Electroacupuncture intervention improves cognitive function of AD model mice. (**A**) Schematic of animal experiment. Schematic depicting the sequence of experimental procedures over days. All mice first undergo a 7-day acclimation. After the acclimation, the EA+Antibiotic group received antibiotic treatment for 2 weeks. After the antibiotic treatment in the EA+Antibiotic group was completed, electroacupuncture intervention was started together with the EA group for 4 weeks. After all the above interventions were completed, behavioral experiments were conducted on all mice. Finally, samples were collected for subsequent biochemical detection. (**B**) Representative escape paths in the Morris water maze. Trajectories of mice in different groups (Control, Model, EA, EA + Antibiotic) during the Morris water maze test, illustrating movement patterns in searching for the hidden platform. (**C**) Escape latency in the Morris water maze (n = 12 per group). Graph showing the escape latency (time to find the platform, in seconds) of mice in each group across 5 test days. Data reflect the ability of spatial learning and memory acquisition. (**D**) Number of platform crossings in the probe trial (n = 12 per group). Bar chart presenting the count of times mice crossed the original platform location in the probe trial of the Morris water maze, indicating memory retention. (**E**) Novel object recognition index (n = 12 per group). Bar graph showing the novel object preference coefficient of mice in each group, a measure of recognition memory in the novel object recognition test. (**F**) Nissl staining of hippocampal subregions (n = 6 per group). Microscopic images of Nissl-stained brain sections, displaying neuronal morphology in the hippocampus and its subregions (dentate gyrus, DG; cornu ammonis 1, CA1; cornu ammonis 3, CA3) for different experimental groups. Scale bar = 100 μm. (**G**) Immunohistochemical staining of hippocampus and cortex regions (n = 6 per group). Images of immunohistochemically stained brain sections, visualizing tau protein expression in the CA1 subregion of the hippocampus and the cerebral cortex across groups. Scale bar = 100 μm. (**H**) Inflammatory cytokine and protein levels in hippocampus and cortex (n = 6 per group). Group of bar charts quantifying the levels of TNF-α, IL-1β, and Aβ_1-42_ in the hippocampus and cerebral cortex. ** *p * <  0.01, *** *p * <  0.001 Statistical analysis: The Wilcoxon rank-sum test was applied to compare the differences in escape latency, number of platform crossings, novel object recognition index, and levels of inflammatory cytokines/proteins between groups. All data are presented as mean ± SD. Statistical significance was set as ** *p* < 0.01, *** *p* < 0.001. “ns” indicates no.

To evaluate the restorative effects of EA on learning and memory functions in AD model, this study employed classical behavioral tests, including the Morris water maze and novel object recognition test, to assess changes in learning and memory performance before and after EA treatment. The experimental design and protocol for EA in AD model are shown in Figure 1A. As expected, EA effectively alleviated cognitive impairment, including declines in learning and memory abilities as well as working memory. In addition, to explore whether EA could maintain their neuroprotective effect in the absence of gut microbiota, antibiotics were utilized before EA treatment administration to deplete the bacteria. Pretreatment with antibiotics was performed following the protocol described in the previous research [36]. Our findings revealed that EA had no significant impact on cognitive function in animals pretreated with antibiotics. This result suggests that the gut microbiota is indispensable for EA’s cognitive protective effect, as antibiotic-induced gut microbiota depletion abolished EA’s beneficial effects, consistent with the gut–brain axis mediating EA’s neuroprotection (Figure 1B–E).

As Aβ, tau, and neuroinflammation have been previously regarded as the core pathological basis of AD [37], we subsequently examined the neuronal damage in the brains of AD model mice. Nissl staining results showed significant neuronal damage in the hippocampal and cortical regions of Model group mice, while EA intervention alleviated this damage (Figure 1F). ELISA technology was further used to detect the levels of pro-inflammatory cytokines and Aβ in the hippocampal and cortical tissues, and immunohistochemical staining was combined to observe the expression of tau protein (Figure 1G). The results showed that compared with the control group, the levels of pro-inflammatory cytokines, Aβ content, and tau protein in the hippocampal and cortical tissues of the Model group were significantly higher, while EA treatment significantly reduced the levels of the above indicators (Figure 1H).

Notably, TNF-α levels exhibited a similar variation pattern to Aβ_1-42_: both were significantly elevated in the Model group compared to the Control group, and significantly reduced after EA treatment. This consistency suggests a synergistic pathological role of TNF-α and Aβ_1-42_ in AD: Aβ aggregation can activate microglia to secrete TNF-α, while TNF-α further promotes Aβ deposition by inhibiting Aβ clearance [38]. In contrast, IL-1β showed an opposite trend in the cortex, which may be attributed to the dual role of IL-1β in AD: in the early stage of neuroinflammation, IL-1β can promote the activation of neuroprotective pathways, while in the late stage, it exacerbates neuronal damage [39]. EA may modulate IL-1β in a region-specific manner to balance its pro-inflammatory and neuroprotective effects. Collectively, EA reduces neuroinflammation and Aβ pathology simultaneously, breaking the “Aβ-inflammation” positive feedback loop and exerting neuroprotective effects.

The above experimental results confirm that the AD model used in this study has clear neuronal damage and core pathological changes in AD, and EA can exert neuroprotective effects by reducing neuronal damage and improving related pathological features. However, these neuroprotective effects were markedly diminished after broad-spectrum antibiotic administration, suggesting that the gut microbiota play an indispensable mediating role in EA therapy.

### 3.2. EA Alters Gut Microbial Diversity to Restore Microbiome Balance in AD Model

**Figure 2:**
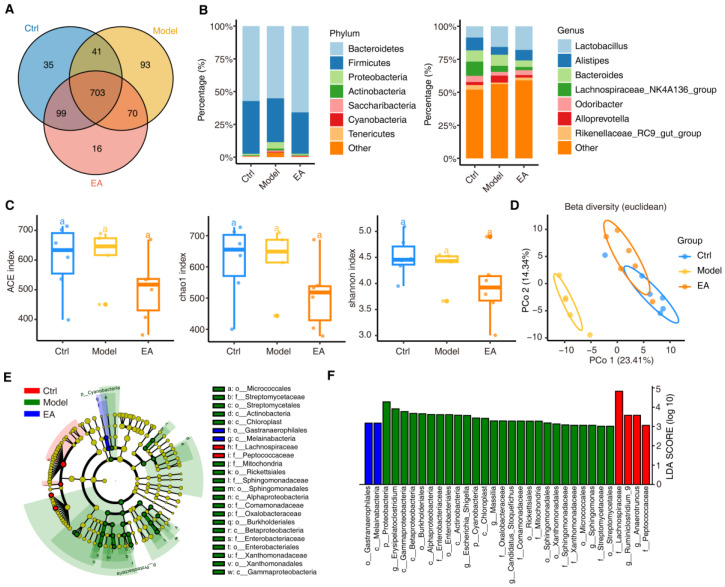
Electroacupuncture alters gut microbial diversity to restore microbiome balance in AD. (**A**) Venn diagram showing the distribution of amplicon sequence variants (ASVs) among the control group (Ctrl, blue), AD model group (Model, yellow), and electroacupuncture-treated group (EA, red). Different colors represent ASVs unique to each group or shared among groups. (**B**) Bar plots illustrating the relative abundance of microbial taxa at the phylum (**left**) and genus (**right**) levels. Colors denote specific taxa, with changes observed across the three groups. (**C**) Box plots of alpha diversity indices (ACE, Chao1, and Shannon) representing microbial richness and diversity across the groups. “a” indicates statistical significance, “line” represents the median, and “cycle” represents a single sample. (**D**) Principal coordinate analysis (PCoA) plot showing beta diversity based on Euclidean distance. Different colors represent the three groups, and ellipses indicate group clustering. (**E**) Cladogram from LefSe analysis showing the phylogenetic distribution of differentially abundant taxa. Colors indicate taxa enriched in each group. (**F**) Bar chart displaying LDA scores from LefSe analysis, identifying taxa with significant contributions to differences among the groups. Statistical analysis: PCA was used to evaluate the clustering trend of samples in metabolomic and microbiomic analyses.

To investigate the impact of EA treatment on the diversity of gut microbiota in a mouse model of AD, we conducted 16S rRNA gene high-throughput sequencing analysis on fecal samples from the control group (Ctrl), AD model group (Model), and EA treatment group (EA). Through OTU clustering, a total of 703 operational taxonomic units (OTUs) shared among the three groups were detected, while unique OTUs were also identified in each group: 35 unique OTUs in the Ctrl group, 93 in Model group, and 16 in the EA group (Figure 2A). The OTU clustering results indicated certain differences in microbial communities among the three groups, with the Model group having the highest total number of OTUs and the most unique OTUs.

Taxonomic annotation revealed the dominant microbial phyla, classes, orders, families, and genera in each group. At the phylum level, the dominant phyla among the three groups were *Bacteroidetes* and *Firmicutes*. The proportion of *Bacteroidetes* was highest in the Ctrl group, averaging 57%, but decreased to about 55% in the Model group. In the EA group, the proportion of *Bacteroidetes* significantly increased to 65%. Additionally, the relative proportion of *Proteobacteria* showed significant differences among the three groups. *Proteobacteria* significantly increased in Model group (from 1% to 5%) but recovered to 0.8% in the EA group (Figure 2B, left). At the genus level, the proportion of *Lactobacillus* was higher in the EA group compared to Ctrl and Model groups. Furthermore, the abundance of *Alistipes* decreased from 9.7% in Model group to 5.9% in Model group, but increased to 8.0% in the EA group. Similarly, *Odoribacter* decreased from 4.7% in Ctrl group to 2.8% in Model group, but increased to 3.7% in the EA group. Other genera such as *Alloprevotella* and *Rikenellaceae*_RC9_gut_group also showed restorative effects following EA treatment (Figure 2B, right). These results suggest that EA treatment can modulate the proportions of key gut microbiota.

Alpha diversity indices are commonly used to describe species richness and evenness within a specific region [40]. We used Alpha diversity indices (including ACE, Chao1, and Shannon indices) to assess the richness and diversity of the microbiota. The results showed that the Alpha diversity of the EA group was significantly lower compared to Ctrl and Model groups (Figure 2C), indicating that EA treatment may selectively enrich beneficial microbiota by reducing the overall diversity of gut microbiota, thereby optimizing the gut microecology.

Additionally, beta diversity analysis, through principal coordinate analysis (PCoA), demonstrated significant differences in microbiota composition among the three groups (Figure 2D). The first two principal coordinates, PC1 and PC2, explained 23.41% and 14.34% of the community variation, respectively, accounting for a total of 37.75% of the variation. The distribution of samples from different groups in the PCoA space reflected significant differences in their microbial community structures. The samples within each group clustered closely in the PCoA plot, indicating good consistency in gut microbiota structure within groups. The Model group (yellow) samples were distinctly separated from the control group along the PC1 axis, suggesting that the AD state led to significant changes in gut microbiota structure, indicating further exacerbation of community structure diversity. After EA treatment (orange), the samples partially returned to the distribution range of the Ctrl group along the PC1 axis, although they still differed from the Ctrl group, they were significantly closer compared to Model group, indicating that EA treatment partially restored the gut microbiota structure. The beta diversity analysis results suggest that EA treatment can partially reverse the microbial community dysbiosis induced by the AD state by influencing microbiota composition.

Subsequently, we further identified microorganisms with significantly different abundances among the three groups through linear discriminant analysis effect size (LEfSe) analysis (Figure 2E,F). The results showed that Model group (green) was significantly enriched with *Proteobacteria* and *Enterobacteriaceae*, suggesting that these microbiota are closely related to a pro-inflammatory state. The Ctrl group (red) was enriched with *Lachnospiraceae* within the Firmicutes phylum, which are typically associated with a healthy state and anti-inflammatory effects. The EA group (blue) was significantly enriched with the order *Gastranaerophilales* and the class *Melainabacteria*, indicating that EA treatment can reduce pro-inflammatory microbiota in the AD model.

In summary, the above results demonstrate that EA treatment can significantly modulate the diversity of gut microbiota in a mouse model of AD, particularly by reducing microbial species richness and optimizing microbial community structure, providing support for EA treatment of AD at the gut microbiota level. Further research will delve into the specific changes in microbiota and their potential functional roles.

### 3.3. EA Restores Gut Microbial and Functional Imbalances in AD Model

To more accurately evaluate the restorative effects of EA treatment on gut microbiota dysbiosis in AD models, we first filtered bacterial phyla and genera with a relative abundance greater than 0.001% to ensure the biological significance of the analysis. Subsequently, the Wilcoxon rank-sum test was used to statistically analyze differences in microbial abundance between Ctrl group and Model group (*p* < 0.05). We further defined the “restoration trend” as a median abundance pattern of “low–high–low” or “high–low–high” changes, identifying microbiota that showed significant alterations in Model group and exhibited restoration trends after EA treatment.

**Figure 3:**
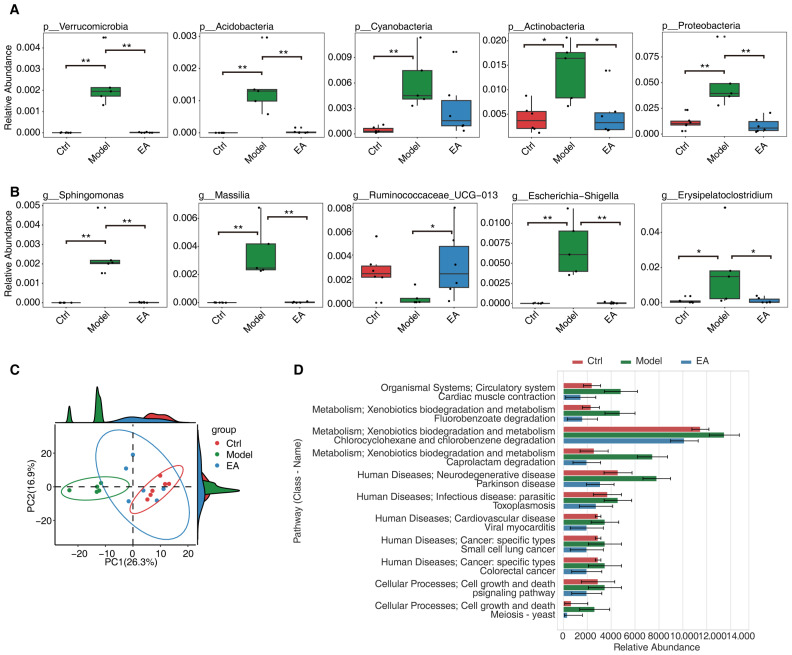
Electroacupuncture restores gut microbial and functional imbalances in AD model. (**A**) Box plots showing the relative abundance of specific bacterial phyla recovered by EA-treatment, including Verrucomicrobia, Acidobacteria, Cyanobacteria, Actinobacteria, and Proteobacteria, across the control group, AD model group, and electroacupuncture-treated group. (**B**) Box plots showing the relative abundance of specific bacterial genera recovered by EA-treatment, including Sphingomonas, Massilia, Ruminococcaceae_UCG-013, Escherichia-Shigella, and Erysipelatoclostridium, across the three groups. The “line” represents the median, and “dot” represents a single sample. (**C**) Principal component analysis (PCA) plot of microbial KEGG pathways showing the clustering of control, model, and electroacupuncture groups. Different colors represent the three groups, with the density distribution of samples along PC1 and PC2 illustrated. (**D**) Bar plot showing the relative abundance of KEGG pathways recovered by EA-treatment across the three groups. Pathways are categorized into metabolism, human diseases, and cellular processes, with distinct patterns observed among the groups. Statistical significance was set as * *p* < 0.05, ** *p* < 0.01.

At the phylum level, we found that the relative abundances of *Verrucomicrobia*, *Acidobacteria*, *Cyanobacteria*, *Actinobacteria*, and *Proteobacteria* were significantly increased in Model group, while their abundances markedly decreased after EA treatment, approaching levels observed in the Ctrl group (Figure 3A). These changes suggest that EA treatment may restore gut microbial ecological balance by modulating the abundance of key phyla.

At the genus level, genera with significant restoration after EA treatment included *Sphingomonas*, *Massilia, Ruminococcaceae_UCG-013, Escherichia-Shigella, and Erysipelatoclostridium* (Figure 3B). Among these, *Ruminococcaceae_UCG-013* was significantly reduced in the Model group but rebounded after EA treatment. Other genera showed abnormal enrichment in the Model group, followed by a significant decline post-EA treatment, aligning with the Ctrl group levels. These results further support that EA therapy selectively regulates the abundance of health-associated genera, alleviating microbial dysbiosis.

To investigate the functional impact of EA treatment on the microbiota, we compared differences in functional pathways across the three groups using principal component analysis (PCA). The results revealed clear separation between the Model group and the Ctrl group along the PC1 (26.3%) and PC2 (16.9%) dimensions, while the EA group partially clustered closer to the control group (Figure 3C). This indicates that EA treatment may partially restore functional pathway states associated with normal gut ecology.

The antibiotic experiment in our study clearly elucidates the dual mechanisms underlying EA therapy: it eliminates or reduces harmful microorganisms and their toxic metabolites, while concurrently promoting the enrichment of beneficial microorganisms and their associated metabolites. This latter process is precisely the critical pathway that antibiotics fail to replicate and may even disrupt. These findings fully demonstrate that EA exerts its effects through the simultaneous action of two indispensable dimensions: “inhibiting pathogenic microorganisms” and “enriching probiotic microorganisms,” with neither being dispensable.

Additionally, to explore the regulatory effects of EA on functional pathways, we conducted KEGG pathway analysis and selected pathways with median abundance changes displaying “low–high–low” or “high–low–high” patterns across the three groups (Figure 3D). The analysis showed that pathways related to neurodegenerative diseases, infectious diseases, and cancer were significantly enriched in the Model group, whereas their abundances decreased notably in the EA group, approaching the Ctrl group levels. These findings suggest that EA treatment may ameliorate pathological gut microecological states by suppressing disease-associated functional pathways.

### 3.4. EA Influences Metabolic Function by Regulating Gut Microbiota

**Figure 4:**
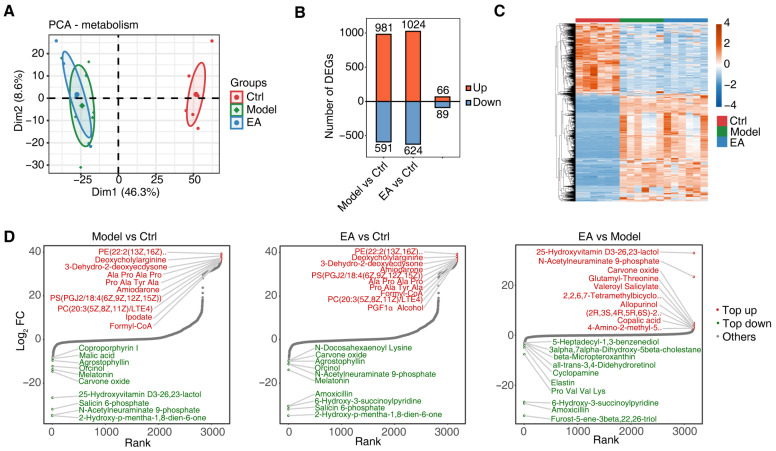
Electroacupuncture influences metabolic function. (**A**) PCA plot of metabolomic data showing the clustering of control, AD model, and electroacupuncture-treated groups. Samples are distributed along principal components (PC1 and PC2), representing variations in metabolomic profiles among groups. (**B**) Bar plot showing the number of differentially expressed metabolites (DEMs) between groups. Red indicates upregulated metabolites, and blue indicates downregulated metabolites. (**C**) Heatmap showing the expression levels of significantly altered metabolites across the groups. The color gradient represents the relative abundance of metabolites, with blue indicating low levels and red indicating high levels. (**D**) Volcano plots ranking differentially expressed metabolites between groups. Red points indicate top upregulated metabolites, and green points indicate top downregulated metabolites. Statistical analysis: PCA was used to evaluate the clustering trend of samples in metabolomic and microbiomic analyses.

Microbiota-derived metabolites influence host physiology and behavior through multiple pathways, and their dysregulation may play a critical role in the pathogenesis of neurological disorders [41]. Our study investigated how EA treatment modulates gut microbiota and their metabolites to affect host metabolic function using metabolomic analysis. Principal component analysis (PCA) revealed significant separation between the Model group and the Ctrl group along the PC1 (46.3%) and PC2 (8.6%) dimensions, indicating pronounced metabolic disturbances in the AD model. The EA group clustered closer to the Model group rather than the Ctrl group (Figure 4A), suggesting limited regulatory effects of EA on host metabolism, possibly through partial modulation of specific metabolites. A comparison of differential metabolites across groups showed a larger number of differences between the Model group vs. Ctrl group and EA group vs. Ctr group, whereas fewer differences were observed between the EA group and Model group (Figure 4B), further supporting that EA may act via selective regulation of key metabolites. The heatmap (Figure 4C) demonstrated that most metabolites were significantly up-regulated in the Model group compared to the Ctrl group, with minor adjustments in metabolite abundance after EA treatment.

We further visualized differential metabolite changes between groups (Model vs. Ctrl, EA vs. Ctrl, and EA vs. Model) based on fold change magnitude (Figure 4D). Compared to the Ctrl group, metabolites such as PE(22:2(13Z,16Z)), Deoxycholylariginine, and PS(PGJ2/18:4(6Z,9Z,12Z,15Z)) were significantly up-regulated in the Model group, while down-regulated metabolites like Melatonin and Coproporphyrin I reflected AD-associated metabolic dysregulation [42]. The EA group exhibited a metabolite profile similar to the Model group when compared to the Ctrl group, indicating incomplete restoration of pathological metabolites to normal levels. However, metabolites such as 25-Hydroxyvitamin D3-26,23-lactol, N-Acetylneuraminate 9-phosphate, and carvone oxide were significantly up-regulated in the EA group compared to the Model group, despite being among the most down-regulated metabolites in the Model vs. Ctrl comparison (Top 10). This suggests that EA may partially restore metabolic function by targeting critical metabolites.

### 3.5. Correlation Analysis of Metabolites and Gut Microbiota and Regulatory Mechanisms of EA

**Figure 5:**
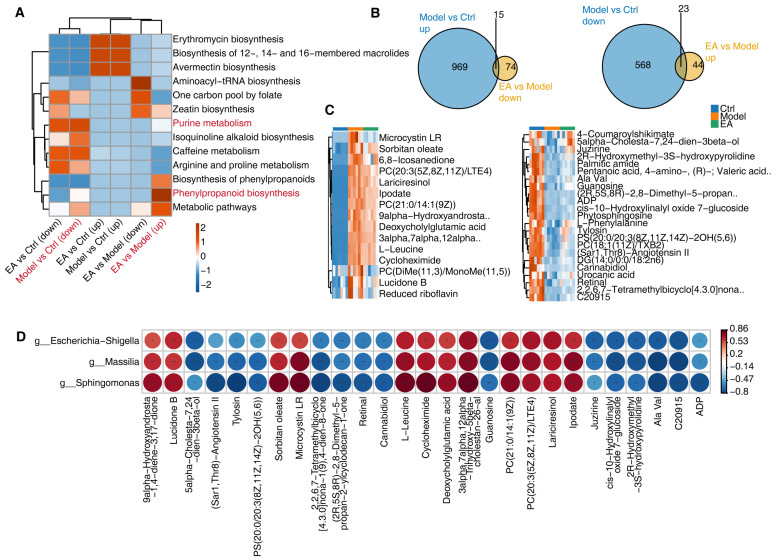
Electroacupuncture affects metabolic function by modulating the gut microbiota. (**A**) Heatmap showing pathway enrichment analysis of differentially expressed metabolites, with pathways categorized into biosynthesis, metabolism, and other functional processes. Colors represent the degree of enrichment. (**B**) Venn diagrams showing the overlap of differentially expressed metabolites. Left diagram represents upregulated metabolites in Model vs. Control and downregulated metabolites in EA vs. Model; right diagram represents the inverse relationship. (**C**) Heatmaps of representative key metabolites recovered by EA-treatment in different groups, illustrating specific metabolite changes associated with electroacupuncture treatment. (**D**) Correlation analysis showing associations between specific metabolites and the abundance of microbial, with significant correlations marked. The shade of the color corresponds to the size of the circle, which means both are associated with the corresponding values indicated in the legend. * indicates *p* < 0.05, ** indicates *p* < 0.01, and *** indicates *p* < 0.001 to the figure legend.

To explore the functional significance of differential metabolites, we performed pathway enrichment analysis using the KEGG database (Figure 5A). Compared to the Ctrl group, the Model group showed significant downregulation of pathways such as phenylpropanoid biosynthesis and purine metabolism, which are closely linked to neuronal function and cerebral metabolism. The downregulation of phenylpropanoid biosynthesis (associated with antioxidant activity) may exacerbate oxidative stress, while impaired purine metabolism (critical for energy production and nucleic acid synthesis) could lead to energy deficits and neuronal dysfunction [43]. EA treatment partially restored these pathways, indicating its potential to alleviate AD-related metabolic disorders by recovering key metabolic functions.

The Venn diagram further illustrates the intersections of differential metabolites. The overlap between up-regulated metabolites in the Model group and down-regulated metabolites in the EA group may represent potential pathological metabolites suppressed by EA treatment. Conversely, the intersection between down-regulated metabolites in the Model group and up-regulated metabolites in the EA group likely signifies key health-associated metabolites restored by EA (Figure 5B). These intersections provide clues to the metabolic regulatory effects of EA. However, in the detailed metabolite analysis, the heatmap reveals that EA has limited restorative capacity for most metabolites. Notably, metabolites with more pronounced recovery include Microcystin LR, PC(DiMe(11,3)/MonoMe(11,5)), Lucidone B, reduced riboflavin, 4-Coumaroylshikimate, 5-alpha-Cholesta-7,24-dien-3beta-ol, and Juzirine (Figure 5C), suggesting that these metabolites may serve as potential therapeutic targets or functional biomarkers of EA.

To further explore the dependencies between gut microbiota and metabolites, we performed Spearman correlation analysis on EA-restored microbes and metabolites, selecting significant pairs with absolute correlation coefficients >0.7 and (*p* < 0.01) (Figure 5D). The results showed that metabolites such as Lucidone B, reduced riboflavin, 4-Coumaroylshikimate, and 5alpha-Cholesta-7,24-dien-3beta-ol were significantly positively correlated with three bacterial genera (Sphingomonas, Massilia, and Escherichia-Shigella), while Microcystin LR, PC(DiMe(11,3)/MonoMe(11,5)), and Juzirine exhibited significant negative correlations. These metabolites may accumulate due to abnormal microbial activity in the pathological state of AD, leading to metabolic imbalances in the nervous system. For example, although Microcystin LR possesses antioxidant properties, its excessive accumulation may increase oxidative stress [44]. EA treatment, by reducing the abundance of these three bacterial genera, may regulate its levels appropriately, thereby mitigating neuronal damage. Similarly, the restoration of metabolic balance in Lucidone B and reduced riboflavin may improve mitochondrial function and energy metabolism, promoting neuroprotection. Additionally, 5alpha-Cholesta-7,24-dien- 3beta-ol, a key intermediate in cholesterol metabolism, plays a crucial role in maintaining neuronal membrane stability.

## 4. Discussion

In this study, we conducted a comprehensive analysis of the role of EA in modulating the gut–brain axis to mitigate AD pathology. To explore the mechanisms underlying EA’s therapeutic effects on AD, we first analyzed its impact on gut microbiota. Subsequently, we integrated metabolomic data to investigate the effects of EA on systemic metabolites. We then performed correlation analyses between gut microbiota and metabolomics to further elucidate the interdependent relationship between gut microbiota and metabolites. A particularly groundbreaking discovery in our study is the positive influence of EA treatment on cognitive function. Through a series of behavioral experiments, we confirmed that EA therapy effectively alleviates cognitive impairment, including declines in learning, memory, and working memory abilities. However, in the absence of gut microbiota, the neuroprotective effects of EA in improving cognitive function were significantly diminished. This finding underscores the crucial role of the gut microbiota in mediating the neuroprotective effects of EA therapy.

Increased permeability of the intestinal and blood–brain barriers, triggered by gut microbiota dysbiosis, elevates the risk of neurodegenerative diseases [45]. AD may originate in the gut and is closely associated with gut microbiota dysregulation. Evidence suggests that gut microorganisms play a role in the evolution of AD pathogenesis [46]. For instance, compared to healthy individuals, AD patients exhibit an increased relative abundance of Bacteroidetes and a decreased abundance of Firmicutes [47]. Recent studies have identified significant gut microbiota dysbiosis in both AD mouse models and patients, which is related to alterations in the abundance and diversity of gut microbes [48]. This implies that the gut bacterial community could be a target for therapeutic interventions in AD. Furthermore, changes in gut microbiome composition have been correlated with Aβ and tau pathological biomarkers, but not with neurodegeneration biomarkers, suggesting that gut microbiota changes may occur in the early stages of the disease [49]. This reinforces the potential utility of gut microbiota as a supplementary biomarker for predicting early-stage disease progression.

EA holds potential for treating nervous system diseases, although its specific underlying mechanisms remain poorly understood, presenting significant challenges to clinical application and management. Specifically, EA alleviates Parkinson’s motor symptoms and dopaminergic neuron damage [50], mitigates postoperative cognitive impairment by enhancing beneficial flora and barrier integrity [51], and improves cognitive deficits in vascular dementia and AD models by correcting microbial imbalances [52]. Treatment regimens at key acupoints like GV20 and BL23 consistently increase cognition-promoting genera and probiotics while reducing inflammatory bacteria and pro-inflammatory cytokines, thereby preventing and treating AD [53,54,55]. Beyond microbiota, EA’s core mechanisms include neuroprotection against oxidative stress, apoptosis, and neuroinflammation; metabolic regulation; modulation of BDNF; and reduction of β-amyloid deposition [56]. Crucially, it inhibits neuroinflammation via pathways like TLR4/NF-κB and NLRP3/caspase-1 [57]. This multi-target approach extends to depression (regulating Bacteroidetes/Firmicutes ratio, neurotransmitters, β-CaMKII/NMDAR/BDNF) [58], IBS (via GDNF signaling), tumor progression (reducing inflammation, enhancing immunity) [59], obesity, and cerebral ischemia, often involving normalization of specific bacterial genera, metabolites like SCFAs, and metabolic pathways (amino acid, purine metabolism) [60], highlighting EA’s broad efficacy through gut–microbiota–brain axis modulation.

To further clarify the key mechanisms of EA’s action, we analyzed the ‘gut microbiota–metabolite–neuron’ regulatory axis. 16S rRNA sequencing showed that EA enriched Gastranaerophilales, which has been associated with short-chain fatty acid (SCFA) production—SCFAs can cross the blood–brain barrier to inhibit neuroinflammation and improve mitochondrial function. Metabolomic analysis revealed that EA restored purine metabolism, and the key metabolite adenosine triphosphate (ATP) was significantly increased in the EA group (*p* < 0.01 vs. Model group). Correlation analysis showed that Gastranaerophilales abundance was positively correlated with ATP levels (r = 0.78, *p* < 0.01), suggesting that EA may promote ATP synthesis via Gastranaerophilales-mediated SCFA production, thereby improving neuronal energy metabolism. Additionally, EA downregulated 5α-cholesta-7,24-dien-3β-ol (a cholesterol metabolite), which was positively correlated with Aβ levels (r = 0.82, *p* < 0.01)—indicating that EA may reduce Aβ aggregation by regulating cholesterol metabolism. These findings link EA’s gut microbiota modulation to specific metabolic and neuronal pathways, providing a more detailed mechanistic basis for its neuroprotective effect.

As we know, glycerophospholipids are the main components of neuronal cell membranes, and their metabolic disorders are closely associated with AD pathology. PC is the most abundant phospholipid in the brain, and its reduction can destroy neuronal membrane fluidity and impair synaptic transmission. In our study, EA reversed the decrease in PC in the Model group, which may improve synaptic plasticity by maintaining membrane integrity. PE is a key precursor for phosphatidylcholine synthesis. EA reduced PE levels to near-normal, which may alleviate mitochondrial damage. PS is involved in Aβ clearance: high levels of PS can promote Aβ aggregation by binding to Aβ peptides. EA’s reduction of PS levels may inhibit Aβ deposition. Collectively, EA modulates the levels of PE, PC, and PS, restoring neuronal membrane function and breaking the pathological chain of AD.

While previous studies focusing on single genes or pathways have shown EA’s potential in alleviating pain and improving certain neurological conditions, our research is the first to systematically elucidate the molecular mechanisms by which EA ameliorates AD through the gut–brain axis via multi-omics integration. Notably, EA significantly restored gut microbiota α/β diversity in AD models, reversed abnormal abundances of pro-inflammatory phyla, such as Verrucomicrobia reduced from 5% in Model group to 0.8% in EA group, and selectively enriched beneficial genera like Lactobacillus. Gut microbiota transplantation experiments confirmed the neuroprotective effects depend on microbial ecology. Furthermore, metabolomics revealed EA restored two core pathways: purine metabolism, which is critical for mitochondrial energy supply, and phenylpropanoid metabolism involved in antioxidant stress, while regulating 12 key metabolites including Microcystin LR and melatonin. Metabolites such as 5α-Cholesta-7,24-dien-3β-ol demonstrated direct correlations with neuronal membrane stability. The constructed “gut microbiota-metabolite” regulatory network has demonstrated how genera such as Escherichia-Shigella target apoptosis-related genes via metabolites. This research innovatively reveals the multidimensional synergistic mechanisms underlying the therapeutic effects of EA on AD pathology, establishing a novel biological foundation for the use of acupuncture in treating neurodegenerative disorders.

In summary, our study innovatively and systematically revealed EA’s potential neuroprotective effects. Undoubtedly, elucidating the multi-level regulatory effects of EA on gut microbiota, metabolites, and the transcriptome is crucial for uncovering the molecular mechanisms underlying its therapeutic potential for AD. However, this study has limitations that need acknowledgment. First, it only utilized the single *SAMP8* AD model. Although this model shares pathological similarities with human AD, it cannot fully replicate the complexity of human AD. Second, the metabolite targets predicted via PubChem/ChEMBL lack in vitro validation (e.g., luciferase assay, Western blot) and thus require further experimental confirmation.

Based on these, future directions include (1) using multiple AD models (*SAMP8*, *APP/PS1*, *5×FAD*) to verify EA’s regulatory effects on different AD pathologies and its efficacy universality; (2) validating key EA-regulated metabolites effects on Aβ aggregation/tau phosphorylation via in vitro cell experiments, and confirming gut microbiota’s mediating role in EA’s efficacy via FMT (transplanting EA-treated *SAMP8* fecal microbiota to untreated *SAMP8* mice); and (3) designing randomized, controlled, double-blind clinical studies with mild-to-moderate AD patients to evaluate EA’s (GV20/BL23 stimulation) effects on cognitive function (e.g., MMSE), gut microbiota, and metabolites for clinical application evidence.

## 5. Conclusions

Our current study revealed that EA significantly improved learning, memory and recognition in AD model mice, reduced neuronal damage, TNF-α and IL-1β, and alleviated tau and Aβ pathologies. EA modulated gut microbiota by enriching beneficial taxa and reducing pro-inflammatory ones, while restoring metabolic pathways, lowering harmful metabolites, and improving oxidative stress and mitochondrial function, thereby exerting neuroprotection. Thus, EA shows potential as a non-pharmacological strategy for AD, though its clinical application requires further validation.

## Data Availability

All data in the current study are available from the corresponding authors upon reasonable request.

## References

[1] Zhang J., Zhang Y., Wang J., Xia Y., Zhang J., Chen L. (2024). Recent advances in Alzheimer’s disease: Mechanisms, clinical trials and new drug development strategies. Signal Transduct. Target. Ther..

[2] Beata B.K., Wojciech J., Johannes K., Piotr L., Barbara M. (2023). Alzheimer’s Disease-Biochemical and Psychological Background for Diagnosis and Treatment. Int. J. Mol. Sci..

[3] Ma Q., Xing C., Long W., Wang H.Y., Liu Q., Wang R.F. (2019). Impact of microbiota on central nervous system and neurological diseases: The gut-brain axis. J. Neuroinflamm..

[4] Megur A., Baltriukienė D., Bukelskienė V., Burokas A. (2020). The Microbiota-Gut-Brain Axis and Alzheimer’s Disease: Neuroinflammation Is to Blame?. Nutrients.

[5] You M., Chen N., Yang Y., Cheng L., He H., Cai Y., Liu Y., Liu H., Hong G. (2024). The gut microbiota-brain axis in neurological disorders. MedComm.

[6] Zhao Q., Chen Y., Huang W., Zhou H., Zhang W. (2023). Drug-microbiota interactions: An emerging priority for precision medicine. Signal Transduct. Target. Ther..

[7] Liu P., Wu L., Peng G., Han Y., Tang R., Ge J., Zhang L., Jia L., Yue S., Zhou K. (2019). Altered microbiomes distinguish Alzheimer’s disease from amnestic mild cognitive impairment and health in a Chinese cohort. Brain Behav. Immun..

[8] Askarova S., Umbayev B., Masoud A.R., Kaiyrlykyzy A., Safarova Y., Tsoy A., Olzhayev F., Kushugulova A. (2020). The Links Between the Gut Microbiome, Aging, Modern Lifestyle and Alzheimer’s Disease. Front. Cell. Infect. Microbiol..

[9] Erny D., Dokalis N., Mezö C., Castoldi A., Mossad O., Staszewski O., Frosch M., Villa M., Fuchs V., Mayer A. (2021). Microbiota-derived acetate enables the metabolic fitness of the brain innate immune system during health and disease. Cell Metab..

[10] Sun B., Cao X., Xin M., Guan R. (2024). Treatment of Depression with Acupuncture Based on Pathophysiological Mechanism. Int. J. Gen. Med..

[11] Zhang B., Shi H., Cao S., Xie L., Ren P., Wang J., Shi B. (2022). Revealing the magic of acupuncture based on biological mechanisms: A literature review. Biosci. Trends..

[12] Ye H.M., Li Z.Y., Zhang P., Kang Z., Zhou D.S. (2025). Exploring Mechanism of Electroacupuncture in Modulating Neuroinflammation Based on Intestinal Flora and Its Metabolites. Chin. J. Integr. Med..

[13] Jia Y., Zhang X., Yu J., Han J., Yu T., Shi J., Zhao L., Nie K. (2017). Acupuncture for patients with mild to moderate Alzheimer’s disease: A randomized controlled trial. BMC Complement. Altern. Med..

[14] Xin Y., Zhou S., Chu T., Zhou Y., Xu A. (2025). Protective Role of Electroacupuncture Against Cognitive Impairment in Neurological Diseases. Curr. Neuropharmacol..

[15] Zheng X., Lin W., Jiang Y., Lu K., Wei W., Huo Q., Cui S., Yang X., Li M., Xu N. (2021). Electroacupuncture ameliorates beta-amyloid pathology and cognitive impairment in Alzheimer disease via a novel mechanism involving activation of TFEB (transcription factor EB). Autophagy.

[16] Xin Y.Y., Wang J.X., Xu A.J. (2022). Electroacupuncture ameliorates neuroinflammation in animal models. Acupunct. Med..

[17] Kong X., Ma Z., Tang R., Wang X., Wei K., Yang G., Yang Y., Zhao Y., Zhang D., Xie C. (2023). Efficacy of acupuncture in patients with mild Alzheimer’s disease and its impact on gut microbiota: Study protocol for a randomized sham-controlled trial. Front. Med..

[18] Zhang Q., Deng P., Chen S., Xu H., Zhang Y., Chen H., Zhang J., Sun H. (2023). Electroacupuncture and human iPSC-derived small extracellular vesicles regulate the gut microbiota in ischemic stroke via the brain-gut axis. Front. Immunol..

[19] Sun X., Zhang A., Pang B., Wu Y., Shi J., Zhang N., Ye T. (2024). Electroacupuncture pretreatment alleviates spasticity after stroke in rats by inducing the NF-κB/NLRP3 signaling pathway and the gut-brain axis. Brain Res..

[20] Jiang J., Liu H., Wang Z., Tian H., Wang S., Yang J., Ren J. (2021). Electroacupuncture could balance the gut microbiota and improve the learning and memory abilities of Alzheimer’s disease animal model. PLoS ONE.

[21] Cai M., Lee J.H., Yang E.J. (2019). Electroacupuncture attenuates cognition impairment via anti-neuroinflammation in an Alzheimer’s disease animal model. J. Neuroinflamm..

[22] Yang S.H., Zhang X.X., Zhong Z.Q., Luo X.X., Wang Y.F., Xiao X.P., Huang Z.Q., Yu S.Y., Sun J.Y., Liu M.J. (2023). Metabolomics Analysis of Electroacupuncture Pretreatment Induced Neuroprotection on Mice with Ischemic Stroke. Am. J. Chin. Med..

[23] Edgar R.C. (2010). Search clustering orders of magnitude faster than, B.L.A.S.T. Bioinformatics.

[24] Callahan B., McMurdie P.J., Holmes S. (2017). Exact sequence variants should replace operational taxonomic units in marker-gene data analysis. ISME J..

[25] Quast C., Pruesse E., Yilmaz P., Gerken J., Schweer T., Yarza P., Peplies J., Glöckner F.O. (2012). The SILVA ribosomal RNA gene database project: Improved data processing and web-based tools. Nucleic Acids Res..

[26] Edgar R.C. (2016). SINTAX: A simple non-Bayesian taxonomy classifier for 16S and ITS sequences. bioRxiv.

[27] Dixon P. (2003). VEGAN, a package of R functions for community ecology. J. Veg. Sci..

[28] McMurdie P.J., Holmes S. (2013). phyloseq: An R Package for Reproducible Interactive Analysis and Graphics of Microbiome Census Data. PLoS ONE.

[29] Segata N., Izard J., Waldron L., Gevers D., Miropolsky L., Garrett W.S., Huttenhower C. (2011). Metagenomic biomarker discovery and explanation. Genome Biol..

[30] Douglas G., Maffei V.J., Zaneveld J.R., Yurgel S.N., Brown J.R., Taylor C.M., Huttenhower C., Langille M.G.I. (2020). PICRUSt2 for prediction of metagenome functions. Nat. Biotechnol..

[31] Kim S., Thiessen P.A., Cheng T., Yu B., Bolton E.E. (2018). An update on PUG-REST: RESTful interface for programmatic access to PubChem. Nucleic Acids Res..

[32] Chambers J., Davies M., Gaulton A., Hersey A., Velankar S., Petryszak R., Hastings J., Bellis L., McGlinchey S., Overington J.P. (2013). UniChem: A unified chemical structure cross-referencing and identifier tracking system. J. Cheminform..

[33] Gaulton A., Bellis L.J., Bento A.P., Chambers J., Davies M., Hersey A., Light Y., McGlinchey S., Michalovich D., Al-Lazikani B. (2011). ChEMBL: A large-scale bioactivity database for drug discovery. Nucleic Acids Res..

[34] Keiser M.J., Roth B.L., Armbruster B.N., Ernsberger P., Irwin J.J., Shoichet B.K. (2007). Relating protein pharmacology by ligand chemistry. Nat. Biotechnol..

[35] Wu T., Hu E., Xu S., Chen M., Guo P., Dai Z., Feng T., Zhou L., Tang W., Zhan L. (2021). clusterProfiler 4.0: A universal enrichment tool for interpreting omics data. Innovation.

[36] Li J., Gao Q., Ma Y., Deng Y., Li S., Shi N., Niu H., Liu X.Y., Cai J. (2022). Causality of Opportunistic Pathogen Klebsiella pneumoniae to Hypertension Development. Hypertension.

[37] Si Z.Z., Zou C.J., Mei X., Li X.F., Luo H., Shen Y., Hu J., Li X.X., Wu L., Liu Y. (2023). Targeting neuroinflammation in Alzheimer’s disease: From mechanisms to clinical applications. Neural Regen. Res..

[38] Cai Y., Liu J., Wang B., Sun M., Yang H. (2022). Microglia in the Neuroinflammatory Pathogenesis of Alzheimer’s Disease and Related Therapeutic Targets. Front. Immunol..

[39] Mendiola A.S., Cardona A.E. (2018). The IL-1β phenomena in neuroinflammatory diseases. J. Neural Transm..

[40] Cassol I., Ibañez M., Bustamante J.P. (2025). Key features and guidelines for the application of microbial alpha diversity metrics. Sci. Rep..

[41] Ahmed H., Leyrolle Q., Koistinen V., Kärkkäinen O., Layé S., Delzenne N., Hanhineva K. (2022). Microbiota-derived metabolites as drivers of gut-brain communication. Gut Microbes..

[42] Poddar M.K., Banerjee S., Chakraborty A., Dutta D. (2021). Metabolic disorder in Alzheimer’s disease. Metab. Brain Dis..

[43] Korczowska-Łącka I., Hurła M., Banaszek N., Kobylarek D., Szymanowicz O., Kozubski W., Dorszewska J. (2023). Selected Biomarkers of Oxidative Stress and Energy Metabolism Disorders in Neurological Diseases. Mol. Neurobiol..

[44] Xiao Y., Hu L., Duan J., Che H., Wang W., Yuan Y., Xu J., Chen D., Zhao S. (2024). Polystyrene microplastics enhance microcystin-LR-induced cardiovascular toxicity and oxidative stress in zebrafish embryos. Environ. Pollut..

[45] Mou Y., Du Y., Zhou L., Yue J., Hu X., Liu Y., Chen S., Lin X., Zhang G., Xiao H. (2022). Gut Microbiota Interact With the Brain Through Systemic Chronic Inflammation: Implications on Neuroinflammation, Neurodegeneration, and Aging. Front. Immunol..

[46] Lista S., Munafò A., Caraci F., Imbimbo C., Emanuele E., Minoretti P., Pinto-Fraga J., Merino-País M., Crespo-Escobar P., López-Ortiz S. (2025). Gut microbiota in Alzheimer’s disease: Understanding molecular pathways and potential therapeutic perspectives. Ageing Res. Rev..

[47] Li H., Cui X., Lin Y., Huang F., Tian A., Zhang R. (2024). Gut microbiota changes in patients with Alzheimer’s disease spectrum based on 16S rRNA sequencing: A systematic review and meta-analysis. Front. Aging Neurosci..

[48] Liu S., Gao J., Zhu M., Liu K., Zhang H.L. (2020). Gut Microbiota and Dysbiosis in Alzheimer’s Disease: Implications for Pathogenesis and Treatment. Mol. Neurobiol..

[49] Verhaar B.J.H., Hendriksen H.M.A., de Leeuw F.A., Doorduijn A.S., van Leeuwenstijn M., Teunissen C.E., Barkhof F., Scheltens P., Kraaij R., van Duijn C.M. (2022). Gut Microbiota Composition Is Related to AD Pathology. Front. Immunol..

[50] Hsu W.T., Chen Y.H., Yang H.B., Lin J.G., Hung S.Y. (2020). Electroacupuncture Improves Motor Symptoms of Parkinson’s Disease and Promotes Neuronal Autophagy Activity in Mouse Brain. Am. J. Chin. Med..

[51] Tang L., Zhang X., Zhang B., Chen T., Du Z., Song W., Chen W., Wang C. (2024). Electroacupuncture remodels gut microbiota and metabolites in mice with perioperative neurocognitive impairment. Exp. Gerontol..

[52] Du K., Yang S., Wang J., Zhu G. (2022). Acupuncture Interventions for Alzheimer’s Disease and Vascular Cognitive Disorders: A Review of Mechanisms. Oxid. Med. Cell Longev..

[53] Weng H., Wang Q., Ye R., Bai Y., Yang H., Xu G., Wang Q. (2023). Anti-oxidative-initiated cognitive impairment amelioration in Alzheimer’s disease model rats through preventive transcutaneous electrical acupoint stimulation. Integr. Med. Res..

[54] He C., Huang Z.S., Yu C.C., Wang X.S., Jiang T., Wu M., Kong L.H. (2021). Preventive electroacupuncture ameliorates D-galactose-induced Alzheimer’s disease-like inflammation and memory deficits, probably via modulating the microbiota-gut-brain axis. Iran. J. Basic. Med. Sci..

[55] Zhang Y.F., Xiong W.N., Ma Y.Y. (2025). Exploring the mechanism of action of acupuncture for Alzheimer’s disease based on the immune-metabolic network perspective. Integr. Med. Discov..

[56] Chen S.Y., Gao Y., Sun J.Y., Meng X.L., Yang D., Fan L.H., Xiang L., Wang P. (2020). Traditional Chinese Medicine: Role in Reducing β-Amyloid, Apoptosis, Autophagy, Neuroinflammation, Oxidative Stress, and Mitochondrial Dysfunction of Alzheimer’s Disease. Front. Pharmacol..

[57] Sun L., Yong Y., Wei P., Wang Y., Li H., Zhou Y., Ruan W., Li X., Song J. (2021). Electroacupuncture ameliorates postoperative cognitive dysfunction and associated neuroinflammation via NLRP3 signal inhibition in aged mice. CNS Neurosci Ther..

[58] Li P., Huang W., Yan Y.N., Cheng W., Liu S., Huang Y., Chen W., Chen Y.P., Gao Y., Lu W. (2021). Acupuncture Can Play an Antidepressant Role by Regulating the Intestinal Microbes and Neurotransmitters in a Rat Model of Depression. Med. Sci. Monit..

[59] Zhang Z., Yu Q., Zhang X., Wang X., Su Y., He W., Li J., Wan H., Jing X. (2021). Electroacupuncture regulates inflammatory cytokines by activating the vagus nerve to enhance antitumor immunity in mice with breast tumors. Life Sci..

[60] Zhang Y., Wang X., Deng D., Gong Y., Chen X., Li J., Hong X. (2025). Integrating 16S rDNA and metabolomics to uncover the therapeutic mechanism of electroacupuncture in type 2 diabetic rats. Front. Microbiol..

